# Individualized Treatment Approaches for Obstructive Sleep Apnea in Older Adults: Beyond Continuous Positive Airway Pressure

**DOI:** 10.20900/agmr20260020

**Published:** 2026-08-21

**Authors:** Nikhil Jaganathan, Varun Goel, Xiaoyue Liu, Wenzhan Song, Younghoon Kwon, Jeongok Logan, William J. Healy

**Affiliations:** 1 Medical College of Georgia, Augusta University, Augusta, GA 30912, USA; 2 Rory Meyers College of Nursing, New York University, New York, NY 10010, USA; 3 College of Engineering, University of Georgia, Athens, GA 30602, USA; 4 Division of Cardiology, University of Washington, Seattle, WA 98195, USA; 5 School of Nursing, University of Virginia, Charlottesville, VA 22904, USA; 6 Division of Pulmonary, Critical Care, and Sleep Medicine, Medical College of Georgia, Augusta University; Augusta, GA 30912, USA

**Keywords:** obstructive sleep apnea, continuous positive airway pressure, mandibular advancement devices, positional therapy, pharmacotherapy, glucagon-like peptide-1 agonists, hypoglossal nerve stimulation

## Abstract

With an aging population and greater prevalence of metabolic disorders, obstructive sleep apnea (OSA) is increasingly observed in older patients. OSA in older adults often occurs in the setting of various comorbidities, such as cognitive, cardiac, and metabolic dysfunction, placing these patients at greater risk. Continuous positive airway pressure (CPAP) is the first-line treatment for OSA in most patients, but adherence with CPAP decreases with increasing age. Current literature on considerations and efficacy of treatment modalities for OSA in older patients is limited. This narrative review summarizes current evidence on CPAP and non-CPAP therapies of OSA in older adults with particular attention to age-specific differences in clinical presentation, diagnostic challenges, and implications for quality of life. Relevant guidelines, clinical studies, and systematic reviews selected from the PubMed and Cochrane databases through April 2026 were summarized and categorized based on type of therapy. Some non-CPAP alternatives appear promising or effective in selected adults for improving metrics of sleep, quality of life, and mitigating comorbidities, but for geriatric-specific comparative evidence remains limited, indirect, observational, or derived from adults substantially younger than the target population. Lifestyle modifications, positional therapy, and oral appliances may yield benefits in older patients comparable to those seen in their younger counterparts, but age-stratified studies are limited. While pharmacotherapy is an area of emerging study, GLP-1 RAs show significant early potential with multifaceted benefits, although supporting studies have been primarily conducted in middle-aged adults. Hypoglossal nerve stimulation has emerged as a prominent OSA treatment alternative with improved adherence compared to CPAP in in small elderly-specific studies. OSA management in older patients is highly nuanced with multifactorial consideration of polypharmacy, comorbidities, and patient preferences to optimize patient outcomes. Larger-scale, prospective studies specific to older patients are warranted to assess treatment efficacy, adherence, and adverse effects in this population.

## INTRODUCTION

Due to the increasing age of the population with greater prevalence of metabolic disorders and obesity, obstructive sleep apnea (OSA) is increasingly observed in older patients. Some studies estimate that OSA has a prevalence of 50% in the older population and is even greater in those older than 80 years [1]. Older patients with OSA less commonly present with prominent somnolence but frequently develop symptoms of insomnia [2]. OSA in older adults often occurs in the setting of various comorbidities, such as cognitive, cardiac, and metabolic dysfunction, placing these patients at greater risk [2]. Moreover, OSA is underdiagnosed in older populations due to difficulty with screening, including poor specificity of screening tools and challenges with in-lab polysomnography [3,4] with important implications for timely treatment and preservation of quality of life in this age group.

A systematic review of studies relevant to geriatric OSA yielded few studies examining treatment of this pathology in older patients and very limited data on frail older patients [3]. Studies focusing on OSA in older patients infrequently stratify patients by age and primarily focus on younger and middle-aged individuals [3]. Comorbidities, including hypertension, stroke, falls, impaired quality of life, frailty, and mortality, are associated with OSA [3]. The higher-risk nature of presentation, with unique considerations for this demographic, warrants further investigation into multiple treatment modalities in the setting of varying patient tolerance, contraindications, and preferences. This review aims to characterize age-specific differences in clinical presentation and diagnostic challenges in older adults with OSA, and their impact on functional outcomes and quality of life, and to summarize available evidence on CPAP and alternative OSA therapies in this population.

## METHODS

Current literature on considerations and efficacy of treatment modalities for OSA in older patients remains limited, particularly in non-CPAP alternatives. With older adults frequently having multiple comorbidities and underrepresented from trials, this narrative review strives to examine a poorly synthesized area of study. This narrative review of OSA therapy in older adults involves selected published practice guidelines, clinical studies, and systematic reviews manually searched and selected from the PubMed and Cochrane Library databases by three reviewers. PubMed and Cochrane Library databases were selected due to broad coverage of literature and high-quality evidence pertinent to OSA diagnosis and management in older adults. Studies were selected based on relevance to diagnosis and management of OSA in older adults. Relevant studies through April 2026 (earliest included study from 2000) were reviewed from March to July 2026, with prioritization of studies published within the past ten years.

Literature was selected based on search terms including “obstructive sleep apnea”, “OSA”, “lifestyle modifications”, “continuous positive airway pressure”, “CPAP”, “positional therapy”, “myofunctional therapy”, “pharmacotherapy”, “GLP-1 receptor agonist”, “surgery”, and “hypoglossal nerve stimulation”. Studies specific to older patients (at least 65 years of age) or those including stratification by age were preferentially selected. Relevant studies were qualitatively evaluated based on study design (with preference for prospective clinical trials and primary studies), risk of bias, sample size, and age distribution. Studies were categorized based on treatment modality. This review discusses mechanisms of therapy, current data, efficacy, indications, safety, and considerations for alternative treatment modalities for OSA in older populations.

### OSA IN OLDER ADULTS: CLINICAL PRESENTATION, DIAGNOSTIC CHALLENGES, AND IMPACT ON QUALITY OF LIFE

Clinical presentation of OSA in older adults often differs from that in younger and middle-aged adults, which can obscure recognition of disease in routine care. While loud snoring and prominent excessive daytime sleepiness are classic features in younger patients, older individuals more frequently report insomnia symptoms, fragmented sleep, nocturia, or nonspecific fatigue rather than overt sleepiness. These atypical complaints are nonspecific and can be multifactorial in nature, with other etiologies including multimorbidity, medication effects, depression, neurodegenerative disorders, and primary sleep disorders, among others, thereby delaying referral and diagnostic evaluation [5–7].

Diagnostic assessment of OSA in older adults is complicated by limitations of widely used screening and risk stratification tools and practical barriers to formal sleep testing. Standard questionnaires such as STOP-BANG and the Epworth Sleepiness Scale (ESS) may have reduced specificity or inconsistent sensitivity in seniors [4,8,9]. While screening and subjective questionnaires like the ESS do not directly convey poor diagnostic capability, they may lead to delay of care or underdiagnosis. Cognitive impairment, fragility, sensory deficits, transportation barriers, and mobility limitations may further hinder completion of in-laboratory polysomnography and contribute to underdiagnosis in the oldest and frailest patients. Similar to OSA, studies have found that patients with sleep bruxism, a closely-linked disease process, also struggle with diagnosis by polysomnography when compared to portable electromyography which may present an alternative medium for diagnosis [10,11]. Home sleep apnea testing may lessen some access barriers, but evidence specific to older and frail populations remains limited, and interpretation can be challenging in the setting of comorbid cardiopulmonary disease [9,12]. Although home sleep apnea testing (HSAT) can help bypass travel and mobility challenges for older patients, reduced-channel diagnostic tools carry notable drawbacks in this demographic. Senior populations often live with complex health profiles—such as underlying heart or lung disease, non-obstructive central events, hypoventilation, concurrent insomnia, or baseline memory and neurological decline. In these clinical scenarios, simplified home monitors often lack the diagnostic depth required and can underreport the true severity of airway obstruction [10]. Full overnight in-laboratory polysomnography remains the gold standard when central respiratory drive issues or overlap sleep disorders are suspected, or when severe cognitive or physical limitations render self-directed home equipment unreliable. When in-lab testing is unfeasible, diagnostic accuracy at home can be enhanced by leveraging caregiver assistance for proper lead placement or utilizing remote, technician-guided setup options [9,12].

These diagnostic challenges have important implications for downstream health outcomes and patient-perceived benefits of therapy. Untreated OSA in older adults is associated with cardiovascular and cerebrovascular disease, mood disturbance, and cognitive dysfunction, falls, functional status, and reduced health-related quality of life, all of which may threaten independence [5,7,12–14]. However, residual confounding and phenotype heterogeneity due to shared risk factors and competing comorbidities complicate interpretation. Conversely, available studies of CPAP and selected alternative therapies in older populations suggest that effective treatment can improve sleep-related symptoms, mood, cognitive function, and aspects of daily functioning. Because many older adults may prioritize improved daily functioning, independence, and daytime performance over objective values such as apnea-hypopnea index (AHI) reduction alone, treatment decisions should be individualized through shared decision-making. While the AHI remains the standard severity metric, AHI alone may fail to capture clinically relevant risk in older populations [1]. Additionally, it is important to consider that some patients may have asymptomatic AHI elevations which warrants a benefit-harm assessment on a patient-case basis. Complementary physiological dimensions such as hypoxic burden, oxygen desaturation duration, arousal burden, and symptom heterogeneity may provide valuable prognostic insight beyond event frequency alone [15]. However, because geriatric-specific validation remains emerging, these novel metrics should be interpreted alongside clinical assessment rather than as established geriatric standards. Clinicians should consider the burden of diagnostic testing, treatment complexity, comorbid conditions, and the likelihood of meaningful quality-of-life improvement when selecting therapy [13,14].

### CONTINUOUS POSITIVE AIRWAY PRESSURE

Continuous positive airway pressure (CPAP) is the first-line treatment for OSA in most patients, promoting sustained patency of the pharyngeal airway and reducing collapsibility. The International Geriatric Sleep Medicine Task Force highlights the importance of considering patient preferences, comorbidities, life expectancy, cognitive status, and functional dexterity for operating therapeutic devices [3]. While most studies evaluating CPAP do not stratify by age, OSA has been evaluated in relation to certain outcomes more prevalent in the older population. Lacedonia et al. (2013) conducted a large-scale cohort study of 800 patients in the sleep laboratory with a high elderly demographic and stratification by age [3,16]. This study found that overlap syndrome of OSA and Chronic Obstructive Pulmonary Disease (COPD) is more prevalent in older OSA patients in comparison to middle-aged and young patients [3,16]. Additionally, CPAP yielded significant improvement in blood gas parameters for overlap OSA/COPD compared to baseline untreated levels [3,16]. CPAP in the setting of OSA has been found to reduce incidence of new vascular events following ischemic stroke [3,17,18]. Of note for older patients, CPAP adherence has been associated with improved mood and cognition, including in Alzheimer’s disease patients [3]. While promising, these findings remain observational in adherent populations and are subject to healthy-user bias.

Although CPAP has demonstrated benefits in alleviating somnolence, mood and cognitive disturbances, and nocturnal symptoms in older patients, adherence with CPAP decreases with increasing age, particularly in adults above age 80 and in patients with comorbid cognitive disorders [2]. In older patients, various barriers to adherence include discomfort, maxillofacial anatomy, dental issues, post-traumatic stress disorder associated with claustrophobia, anxiety, memory dysfunction, and dexterity [19]. To maximize CPAP adherence before declaring treatment failure, clinicians should first deploy practical troubleshooting strategies tailored to older patients. Key measures include screening for cognitive, visual, or dexterity limitations, choosing simplified interfaces, and managing nasal dryness with heated humidification. Adherence can be further enhanced through early clinical follow-up, telemonitoring for mask leak, behavioral support, and caregiver training for device assembly [14]. Several non-CPAP therapies show evidence of benefit in selected OSA populations, but data specific to older and frail adults remain limited.

### LIFESTYLE MODIFICATIONS

Lifestyle modifications, including diet and exercise, can have profound impacts on OSA. A cohort study of 690 subjects revealed that 10% weight gain is associated with a 32% increase in OSA severity as measured by apnea-hypopnea index (AHI) [20]. The American Heart Association (AHA) recommends weight loss to be considered for all OSA patients, and 10% weight loss lowers AHI by 26% [20]. Dose-dependent relationships between AHI and weight loss have been established [21]. However, while diet and exercise modification yield modest improvements in AHI, risks from medical and pharmacotherapy-induced weight loss should be considered, and older patients may not tolerate aerobic exercise and weight loss to the same extent as their younger counterparts [20]. In older adults, weight-management interventions should be balanced by a holistic nutritional assessment including ensuring adequate protein intake, resistance exercise, and monitoring of muscle strength given the risk of lean-muscle mass and sarcopenia. Exercise tolerability should also be determined with risk-stratification with regards to frailty and functional status rather than age alone [22]. Furthermore, lifestyle interventions operate through distinct physiological mechanisms and should not be considered interchangeable. Dietary weight loss reduces anatomical upper-airway collapse by lowering parapharyngeal fat burden, whereas exercise training (aerobic conditioning or resistance training) decreases OSA severity through fluid-shift attenuation and systemic anti-inflammatory pathways independent of weight change [23]. Exercise has also been shown to mitigate biochemical markers of inflammation and senescence, offering added protection from comorbid frailty and functional limitations [24]. Additionally, pulmonary or cardiorespiratory conditioning enhances respiratory muscle endurance and cardiovascular reserve, while targeted upper-airway training (myofunctional therapy) specifically strengthens pharyngeal, tongue, and facial muscle tone to prevent neuromuscular collapsibility during sleep which can often be used as an adjunct treatment option for OSA [25]. Protocols require intensive daily commitment (20–30 min daily for 3–6 months) and active motor learning, which presents significant adherence challenges for older adults with cognitive decline, neurological disorders, edentulism, or reduced motor skill acquisition [26]. Myofunctional therapy has been shown to reduce AHI by 50% in adults and 62% in children, with improvements in snoring, daytime sleepiness (assessed by ESS), and nadir oxygen saturation in adults, but efficacy in older populations is uncertain [25]. While promising, this data should be interpreted with caution as pooled data of seniors can demonstrate considerable heterogeneity in reported results. Additionally, geriatric-specific trials evaluating both standalone and combination approaches with CPAP or other non-CPAP therapies remain sparse [27].

### ORAL APPLIANCES

Personalized mandibular advancement devices (MAD) are a common oral appliance for OSA treatment in older patients. These devices maintain the mandible in an advanced position relative to the maxilla to lower airway collapse [28]. A meta-analysis by Sharples et al. (2016) of 77 randomized controlled trials in adult patients found improvements in AHI with both MAD and CPAP with demonstrated clinical efficacy, but mean AHI and ESS scores were lower, implying more effective therapy for OSA with CPAP compared to MAD [29]. While CPAP was more effective in OSA treatment, MAD was determined to be viable in patients who do not tolerate CPAP, and results may be similar to CPAP in mild OSA. While AHI reduction may not be as substantial, patient-reported outcomes may be consistent between MAD and CPAP due to increased adherence [29].

Longer-term studies have also yielded promising findings. Although primarily analyzing middle-aged male patients, Yu et al. (2023) demonstrated significant reductions in the ESS by −3.99 and AHI by −16.77, with sustained effectiveness after five years with potential benefits on blood pressure and quality of life [30]. Some evidence suggests no difference in success rates or extent of bite changes between younger and older patients due to MAD [31]. Awadalla et al. (2025) conducted a retrospective cohort study of 66 patients 65 years of age or older diagnosed with OSA treated with oral appliances [32]. Oral appliance therapy was offered based on patient preference (n = 28), CPAP intolerance (n = 32), or as an alternative therapy (n = 4) [32]. Daytime sleepiness, as assessed by ESS, was reduced in patients with both mild-to-moderate and severe OSA with at least 50% reduction in AHI in 50% of patients [32]. These results highlight tolerable and positive OSA outcomes specifically in older patients.

Disadvantages of MAD include the cost of individualizing the therapy based on the patient’s dental anatomy and adverse effects such as temporomandibular joint dysfunction, mucosal irritation, altered salivation (xerostomia or hypersalivation), transient or permanent occlusal changes, dental migration, and compromised device retention [28]. These side effects are particularly relevant in older adults with pre-existing periodontal disease or dental fragility. Furthermore, while complete or partial edentulism traditionally complicates device stability due to a lack of anatomical anchors, it is not an absolute contraindication; modified appliances such as tongue-retaining devices or implant-supported mandibular advancement solutions can be considered in carefully selected cases [33]. While most MAD trials do not stratify by age, benefits and risks demonstrated in these studies should be considered in older candidates, especially in those intolerant to first-line therapy, and a personalized, patient-centered treatment approach is warranted.

### POSITIONAL THERAPY

Positional therapy for OSA involves preventing OSA patients from supine positioning during sleep, often requiring patients to maintain lateral positioning, preserving airway patency [34]. This treatment option serves as a noninvasive modality which may include backpacks, pillows, alarms, or lumbar binders to maintain sleep position [34]. While positional therapy may have improved adherence compared to CPAP, no significant differences in cognitive outcomes, quality of life, or long-term efficacy have been found [34]. With comparable reductions in AHI as CPAP, positional discomfort, potentially attributed to arthritis, may hinder adherence in older patients [20]. Positional OSA is broadly defined by an overall AHI driven predominantly by supine sleep, though diagnostic criteria vary widely across clinical literature. Relying solely on a supine-to-nonsupine AHI ratio (commonly ≥2) is often insufficient if the nonsupine AHI remains clinically elevated. Furthermore, accurate classification depends heavily on total time spent in each sleeping position and can be confounded by night-to-night position variability [35]. A cross-sectional study by Ann et al. (2023) found that older OSA patients are more likely to experience severe positional OSA that may be more effectively managed with positional therapy [36]. Specifically, 54.6% of older patients versus 31.7% of younger patients demonstrated a supine-to-nonsupine AHI ratio ≥ 10 with nonsupine AHI < 5 events/h, highlighting the prevalence of positional OSA with age and that positional therapy may be an effective alternative or adjunctive therapy in this demographic [36]. While age seems to be an important factor, other factors such as body habitus, craniofacial structure, and sleep architecture may play significant roles in determining positional OSA severity [36]. Similar benefits have been demonstrated between younger and older patients for vibrotactile positional therapy, with improved sleep quality (assessed by ESS and Pittsburgh Sleep Quality Index) and snoring [37]. Beyond maintaining avoidance of supine sleep, head position and elevation are critical, but addressing these components of sleep positioning is complex, and further assessment is warranted to optimize positional therapy. Despite the possible stated benefits, forced lateral positioning introduces age-specific safety and feasibility concerns. Sustained side-sleeping can trigger shoulder/hip osteoarthritis pain, elevate pressure ulcer risk, and impede bed mobility, while night-to-night device management complicates nocturia, increasing fall risk in frail or cognitively impaired adults [34]. Furthermore, while head elevation improves airway collapsibility, it operates via distinct biomechanical mechanisms and requires independent validation.

### PHARMACOTHERAPY

Pharmacotherapy is an area of emerging study, including drugs of varying mechanisms of action, including glucagon-like peptide-1 agonists (GLP-1 RAs), noradrenergic and antimuscarinic agents, cholinergic agents, and more. While pharmacotherapy studies specific to older patients or stratifying by age are limited, analyzing broader data sets and medication safety can yield insights for older populations. Tirzepatide is one the most well-studied pharmacotherapeutic agents for OSA, approved by the Food and Drug Administration (FDA) in December 2024 for moderate and severe OSA in adults with obesity [38]. These agents reduce adiposity surrounding pharyngeal structures by delaying gastric emptying, reducing appetite, lowering inflammation, and altering insulin and glucagon signaling [39]. The SURMOUNT-OSA trial assessed efficacy of the GLP-1 agonist tirzepatide for OSA across two 52-week randomized controlled trials comparing tirzepatide to placebo (mean age of 51.7 years) [40]. The study found that patients receiving tirzepatide had reduced hypoxic burden, systolic blood pressure, high-sensitivity C-reactive protein (hsCRP) concentration, and body weight, with improved patient-reported sleep outcomes [40]. Moreover, these medications may carry cardiovascular benefits for those with atherosclerosis and diabetes, reduce adverse cardiac events, and possibly reduce cognitive decline, but may carry a side-effect profile including but not limited to mild gastrointestinal discomfort, dehydration, sarcopenia, hypotension, and medication interactions [41]. Therefore, these agents may have benefits in elderly patients with OSA and obesity, although studies specific to older populations should be conducted with consideration of frailty and safety profiles.

Various drug types have been investigated through mechanisms related to increased pharyngeal muscle tone, preventing respiratory collapse, including noradrenergic-antimuscarinic combinations, serotonergic agents, and cholinergic agents [42–44]. However, an array of combinations has been evaluated with notable variation in efficacy with very limited clinical evaluation and small sample sizes [42–44]. Additionally, certain agents, such as anticholinergic agents, carry unwanted anticholinergic effects, particularly in older patients who are at added risk of delirium, cognitive decline, constipation, urinary retention and falls.

Sulthiame is an emerging pharmacological agent for treating OSA with promising initial results. Sulthiame is a carbonic anhydrase inhibitor which targets OSA through reducing loop gain, aiding ventilation, and increasing upper airway muscle activity. The multicenter FLOW phase 2 trial assessed sulthiame in 535 adults with mean age 56.1 years, with dose-dependent reductions in daytime somnolence, nocturnal hypoxia, and AHI [45]. The most common side effects included dose-dependent paresthesia, nasopharyngitis, and headache, but it was overall well tolerated [46]. While early studies have shown sulthiame’s safety across broad patient demographics, longitudinal evaluation of effectiveness and adverse effects is needed specifically in older populations. Furthermore, since this emerging data comes from a phase 2 trial, preliminary efficacy and tolerability can only be studied without supporting routine use alongside other therapies discussed thus far.

### SURGICAL THERAPY

Surgery is a treatment option for OSA in patients who have failed alternative therapies, particularly in older patients with higher complication risks, comorbidities, and longer recovery times [46]. In a 2019 analysis of National Inpatient Sample and Nationwide Ambulatory Surgery Sample databases in the United States, approximately 0.03% to 0.77% of all adult OSA patients receive surgical treatment annually, comprising 18,526 cases total, including 12,938 palate surgeries, 3054 hypoglossal nerve stimulation implantations, 1840 maxillomandibular advancements, and 1350 hypopharyngeal surgeries [46]. While surgical treatment for OSA is significantly less common in older patients, exact data regarding prevalence in this population is limited and may lack clear guidelines on indications, invasiveness, and evidence-based expected benefit to patients.

Surgical therapy for severe OSA is highly varied and personalized, including maxillomandibular advancement, adenotonsillectomy, hyoid myotomy, uvuloplasty, genioglossus advancement, and uvulopalatopharyngoplasty [47]. While surgical options are often not curative, clinical outcomes may be improved, with benefits including cardiovascular benefits, mortality, and quality of life varying significantly depending on which type of surgical therapy is pursued [20]. Bariatric surgery can be considered in patients with BMI of at least 35 kg/m^2^, which may facilitate positive metabolic outcomes in addition to improved OSA, but occurrence in older patients is uncommon, due to risks such as chronic emesis and acid reflux, operative risk, sarcopenia, nutritional status, expected benefit, and metabolic disease [20]. Ultimately, anesthesia-associated complications and postoperative pain are critical considerations, especially in older adults, and precise evaluation of benefits and risks is needed [20].

### HYPOGLOSSAL NERVE STIMULATION

Hypoglossal nerve stimulation is an invasive treatment modality recommended for moderate to severe OSA in patients intolerant to CPAP, typically with BMI less than 32 kg/m^2^, but this criteria can depend on jurisdiction, device labelling, insurer policy, and clinical context [20]. Hypoglossal nerve stimulation utilizes an implanted device to stimulate the genioglossus muscle, promoting muscle contraction, anterior tongue movement, and avoidance of airway obstruction [48]. Hypoglossal nerve stimulation has emerged as a prominent OSA treatment alternative, especially in older patients with barriers to adherence [19]. Although older patient populations were identified to have greater incidence of cognitive dysfunction and less social support, which affected device usage, Joo et al. (2025) found that patients reported improved tolerance and adherence compared to CPAP, with resultant improvement in sleep and life quality [19]. Although patients reported subjective usage benefit when compared to their prior CPAP attempts, device usage and adherence may be confounded between the two different types of therapies by factors such as follow-up intensity and patient selection (given they had all already failed CPAP therapy). The most common adverse effects reported were headache, anxiety, and dry mouth, which were comparable between older and younger patients [19]. Other studies, however, have found that surgical pain and residual discomfort, device migration or malfunction, infection risk, and possible future need for revisions are important considerations as well [49]. Zhu et al. (2018) evaluated patients older than 64 years who received hypoglossal nerve stimulator implantation matched with patients younger than 65 years [50]. No significant difference in implantation time, AHI, oxygen desaturation index, or ESS scores was found, and no serious adverse events occurred [50]. Notably, women and older patients may be more responsive to hypoglossal stimulation [20].

In a survey study by Dzierzewski et al. (2024), self-reported questionnaires in adults greater than 50 years undergoing consultation for hypoglossal nerve stimulation (mean age 63.3 years) found 88% of patients having insomnia, 30% having physical functioning difficulties, and 36% having daytime impairments resulting from disturbed sleep [51]. This study found that older patients with untreated OSA may have disproportionately greater cognitive dysfunction, mood disturbances, and functional impairments compared to the overall population, which may warrant greater need for intervention [51]. Further investigation would be necessary to directly compare therapeutic effectiveness rather than reported symptomatic burden.

### LIMITATIONS

As evidence continues to develop in this field of geriatric medicine, the synthesis of guideline-based recommendations will improve. This review is limited by several factors which highlight the current shortcomings of the existing literature. Although we have aimed to review the studies from the lens of older patients, it may be more prudent to investigate OSA therapies and outcomes in patients stratified by functional status, biological age, and frailty rather than chronological age. Given the limited source of studies to aggregate, it was not possible to stratify older patients by classes of age such as 65–74, 75–84, and 85+ which could better characterize challenges faced by therapeutic options as patients age. Other factors which pose considerable effect on therapeutic efficacy include cognitive capacity and caregiver support, both of which are infrequently reported in such studies. It is also important to distinguish between efficacy, tolerability, feasibility, and acceptability when discussing therapeutic options for older patients. While some therapies may be better at reducing AHI, cognitive or physical limitations may limit their feasible application for patients. Our discussion has primarily assessed studies which were able to report quantitative results of efficacy, however many studies, such as those investigating pharmaceutical alternatives/adjuncts to CPAP, are limited by being smaller, short-term trials which evaluate surrogate endpoints rather than functional outcomes and long-term safety and are thus more heterogeneous in nature. Further investigation is warranted for combination therapies of CPAP with the alternatives we have reviewed, which could also offer better insight into direct comparison which is currently lacking. The current level of evidence in the reviewed studies largely have indirect or extrapolated application to the study of OSA treatment in geriatric populations. Table 1 displays a central evidence table which discusses the notable studies included in this review, with specific limitations of each provided in brief.

## CONCLUSIONS

Given the high prevalence of OSA in older adults, the age-specific diagnostic challenges, and its substantial impact on cognitive, cardiovascular, and functional outcomes, improving the understanding of OSA screening and management is of utmost importance. As older patients are at increased risk for onset and exacerbation of cardiovascular and cerebrovascular pathologies due to OSA, tailored therapeutic approaches should be utilized beyond typical treatment modalities, including telemonitoring and patient education, yet the extent of these options on cardiovascular and cerebrovascular disease is limited [2]. With high prevalence of comorbidities, such as coronary artery disease, atrial fibrillation, and dementia in poorly treated OSA, consideration of multiple treatment modalities is critical, especially given progressive decline in CPAP adherence with age [8]. While more invasive therapies, such as surgery, may warrant caution in older patients, other alternative treatment modalities, including lifestyle modifications, oral appliances, positional therapy, pharmacotherapy, and hypoglossal stimulation, each have varying benefits in improving metrics of sleep, quality of life, or mitigating comorbidities making them reasonable considerations for carefully selected patients, although direct comparative and combination-therapy studies and geriatric-specific evidence remains limited. Figure 1 highlights an algorithmic approach to individual OSA treatment with considerations for older populations. Conversely, goals of care and deescalation of care should be applied in older adults where appropriate, with more simplified treatment approaches, symptom management, or observation utilized appropriately, with more simplified treatment approaches, symptom management, or observation utilized appropriately.

Current strength of evidence is indirect and heterogenous across treatment modalities, particularly in older adults, with limited age-specific evaluation. Larger-scale, prospective studies specific to older patients are warranted to assess treatment efficacy, adherence, and adverse effects in this population. Additionally, future studies should stratify by frailty, sex, obesity, OSA phenotype, and comorbidities. In order to more adequately reflect clinical practice, consistent reporting of treatment adherence, more robust safety outcomes, and multiple modalities in conjunction should be evaluated. Moreover, novel diagnostic modalities, including age-specific questionnaires, sleep testing, and prognostic markers, are warranted in this unique population [2]. Ultimately, OSA management in older patients is highly nuanced, requiring multifactorial consideration of polypharmacy, comorbidities, and patient preferences to optimize not only clinical outcomes but also health-related quality of life.

Table 1 synthesizes the central evidence that was notable in this narrative review. Studies were identified by the treatment modality they primarily investigated, study design, sample size, mean age of participant, significant biases or weaknesses, and applicability of conclusions to geriatric recommendations for OSA treatment, which was categorized as either a) Directly applicable, b) Partially indirect, or c) Primarily extrapolated based on the strength of the study design and level of evidence provided.

## Figures and Tables

**Figure 1.**
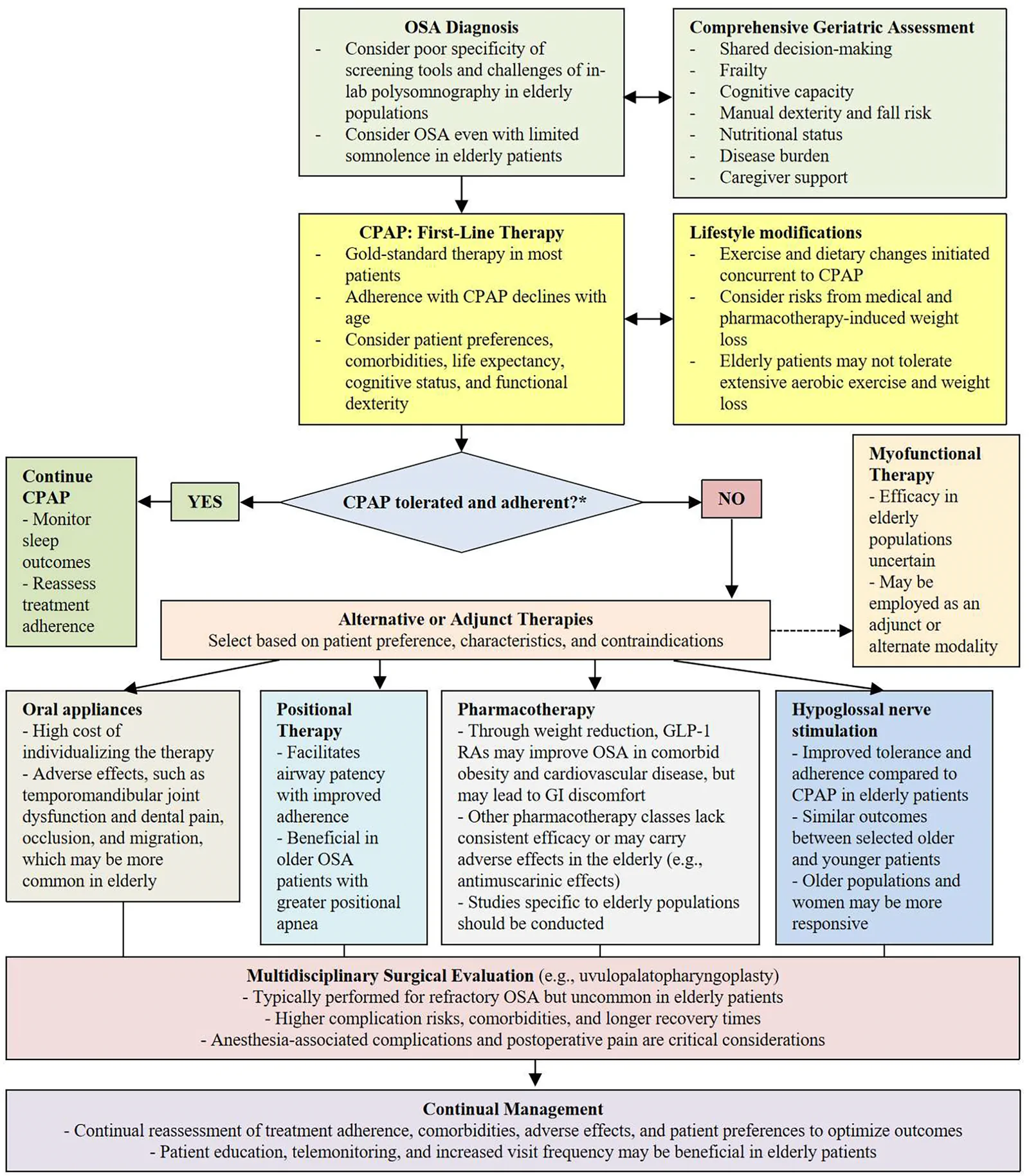
Obstructive Sleep Apnea Treatment and Considerations in Older Adults: A Conceptual Framework. *In addition to CPAP nonadherence, treatment selection depends on OSA severity, symptoms, anatomy, phenotype, obesity, positional dependency, dentition, central-apnea burden, comorbidities, goals, and access to care.

**Table 1.** Central Evidence Table Synthesizing Notable Studies Investigating OSA Treatment Modalities.

Study	Treatment Modality	Study Design	Sample Size	Mean Age	Notable limitations/weaknesses	Direct/Indirect/Extrapolated
[16]	CPAP	Retrospective observational cohort study	720 patients total (OSA = 466, Overlap Syndrome = 168, COPD = 86; sub-analysis on CPAP compliance in 90 patients)	62 years old overall	Investigated a broad age demographic of adults ≥ 18 with little stratification of results by age.	Partially indirect (Substantial older demographic; provided insight into overlap syndrome with COPD, but was not exclusively an age-restricted geriatric trial).
[20]	Lifestyle modification: Weight loss	Prospective, population-based cohort study	690 participants	46 years at baseline	Does not directly assess older patients in comparison with younger patients. Does not stratify patients by functional status or frailty. All participants were employed and ablebodied.	Primarily extrapolated (While foundational for establishing dose-response relationships between weight change and AHI, the data are extrapolated when applied directly to geriatric or frail older adult populations).
[25]	Lifestyle modification: Myofunctional therapy	Systematic review and meta-analysis	145 patients total across 11 studies (9 adult studies with 120 patients; 2 pediatric studies with 25 patients)	44.5 years in the adult studies	The objective of this review was to evaluate myofunctional therapy as a treatment for OSA across all age groups with no specific consideration for older/frail patients.	Primarily extrapolated (Synthesized studies in general adult and pediatric populations without age-stratified geriatric subgroups or specific evaluation of frail older adults)
[29]	Oral appliances	Systematic review and network meta-analysis of randomized controlled trials	77 randomized controlled trials (comprising over 4000 adult participants across CPAP and MAD arms)	Mean ages generally ranged from 45 to 58 years across individual RCTs	There were no direct CPAP vs MAD trials. Primary endpoints were AHI and ESS scores only with no reported functional or patient-centered outcomes.	Primarily extrapolated (The pooled RCT data were derived from general/middle-aged adult trial cohorts without dedicated age-stratified analyses or isolated evaluation of older or frail adults)
[30]	Oral appliances	Systematic review and meta-analysis	546 participants across 20 studies included in the meta-analysis	Mean ages generally ranged from 49 to 62 years across individual studies	Individual studies analyzed had small sample sizes (<30 on average) and high drop-out rates. Primary endpoints were AHI and ESS scores only with no reported functional or patient-centered outcomes.	Primarily extrapolated (Evaluated long-term stability and efficacy of MADs in general adult populations without age-stratified subgroup analyses or specific evaluation of geriatric/frail cohorts)
[32]	Oral appliances	Retrospective cohort study	66 patients total (58 with mild-to-moderate OSA, 8 with severe OSA)	Exact mean age not provided. Baseline age range restricted to adults 65 and older	Primary endpoints were AHI and ESS scores only with no reported functional or patient-centered outcomes.	Directly applicable (Exclusively evaluated patients aged 65 years and older receiving oral appliance therapy for OSA)
[36]	Positional therapy	Cross-sectional observational study	346 patients	67.8 years in the older adult cohort	Does not directly compare positional therapy vs CPAP in older patients. Does not consider other factors outside of supine-to-nonsupine AHI scores in determining positional OSA.	Directly applicable (Exclusively designed to compare age-stratified cohorts and evaluate the specific prevalence and severity of positional OSA in older adults aged 65 and older vs. younger patients)
[40]	Pharma cotherapy	Phase 3, double-blind, randomized, placebo-controlled trials	469 participants across two trials (Trial 1: 234 participants not on CPAP; Trial 2: 235 participants on CPAP)	51.7 years old across both trials	Does not focus on geriatric populations with a mean participant age < 65.	Primarily extrapolated (Evaluated middle-aged adults with obesity and moderate-to-severe OSA; while a small minority of older participants were included, the study was not age-stratified or powered specifically for geriatric or frail populations)
[45]	Pharma cotherapy	Phase 2 Multicenter, randomized, double-blind, placebo-controlled trial	298 patients randomly assigned to placebo and three treatment groups	56.1 years old	Since this emerging data comes from a phase 2 trial, preliminary efficacy and tolerability can only be studied without supporting routine use. Primary objective was to determine safe and effective dosing, not measuring outcomes.	Primarily extrapolated (Evaluated general adult populations aged 18–75 with a mean age around 56 years; excluded adults aged 75 years or older and was not age-stratified or evaluated specifically in geriatric or frail cohorts)
[46]	Surgical therapy	Cross-sectional nationwide database study	18,526 estimated surgical encounters including 12,938 palate surgeries, 3054 hypoglossal nerve stimulation implantations, 1840 MMA procedures, and 1350 hypopharyngeal surgeries	Broad adult population; age stratified across cohorts	Primary objective of this study was to investigate pricing/cost of these surgeries, not functional outcomes for OSA patients. The study was not aimed at elucidating trends in older patients versus younger patients. There were no comparisons made between different surgical therapy options, which varied widely.	Partially indirect (Evaluated nationwide surgical practice patterns and utilization across all adult age groups; provides age-stratified procedural odds such as increased HNS utilization in adults over 40, but not exclusively focused on a dedicated geriatric population).
[19]	Hypoglossal nerve stimulation	Retrospective cohort study/chart review	66 patients total (43 geriatric patients aged 65–86 years compared with 23 younger controls)	66 years old in older cohort	Use of cognitive-behavioral therapy to help mitigate side effect of insomnia in patients may skew reported adherence and efficacy.	Directly applicable (Exclusively focused on comparing real-world clinical implementation, barriers, adherence, and outcomes in patients aged ≥65 years vs. a younger cohort undergoing hypoglossal nerve stimulation).
[50]	Hypoglossal nerve stimulation	Retrospective observational cohort study	62 patients split between older and younger cohorts.	70 years old in study group (patients > 64 years old with HNS device)	Primary endpoints included AHI and ESS scores as well as oxygen desaturation index values, but no functional outcomes or patient-centered outcomes were gathered or reported.	Directly applicable (Evaluated an adult sleep clinic cohort with a wide age range up to 79 years, providing valuable long-term adherence and side-effect data that includes older adults with direct comparison with BMI, AHI matched younger controls.
[51]	Hypoglossal nerve stimulation	Cross-sectional observational study	113 patients	63.3 years old	These patients were actively seeking consultation regarding HNS and therefore were biased against CPAP therapy (presumably already failed). The questionnaire was not designed to evaluate post HNS outcomes.	Partially indirect (Exclusively focused on characterizing older/aging adult candidates seeking hypoglossal nerve stimulation, specifically evaluating age-relevant clinical, functional, cognitive, and psychosocial symptomatic burden only.

## Data Availability

No data were generated from the study.

## ABBREVIATIONS

OSA: obstructive sleep apnea
CPAP: continuous positive airway pressure
MAD: mandibular advancement devices
AHI: apnea-hypopnea index
ESS: Epworth sleepiness scale
GLP-1 RAs: glucagon-like peptide-1 agonists
